# Comprehensive Analysis of Prognostic and Immune Infiltrates for E2F Transcription Factors in Human Pancreatic Adenocarcinoma

**DOI:** 10.3389/fonc.2020.606735

**Published:** 2021-02-02

**Authors:** Xu-Sheng Liu, Yan Gao, Chao Liu, Xue-Qin Chen, Lu-Meng Zhou, Jian-Wei Yang, Xue-Yan Kui, Zhi-Jun Pei

**Affiliations:** ^1^ Department of Nuclear Medicine and Institute of Anesthesiology and Pain, Taihe Hospital, Hubei University of Medicine, Shiyan, China; ^2^ Medical Imaging Center, Taihe Hospital, Hubei University of Medicine, Shiyan, China; ^3^ School of Graduate, Hubei University of Medicine, Shiyan, China; ^4^ Hubei Key Laboratory of Embryonic Stem Cell Research, Shiyan, China; ^5^ Hubei Key Laboratory of Wudang Local Chinese Medicine Research, Shiyan, China

**Keywords:** pancreatic adenocarcinoma, E2F transcription factors, prognostic value, bioinformatics analysis, tumor microenvironment

## Abstract

**Background:**

E2F transcription factors (E2Fs) are a group of genes encoding a family of transcription factors in higher eukaryotes. They are involved in a variety of cellular functions and are up-regulated in many tissues and organs. However, the expression level, genetic variation, molecular mechanism, and biological function of different E2Fs in PAAD and its relationship with the prognosis and immune infiltration in patients with PAAD have not been fully elucidated.

**Methods:**

In this study, we investigated the mRNA expression level, genetic variation, prognostic value and gene–gene interaction network of E2Fs in PAAD using the Oncomine, GEPIA, Kaplan Meier plotter, cBioPortal, GeneMANIA, STRING and Metascape database. Then, the relationship between E2Fs expression and tumor immune invasion was studied by using the TIMER database. Finally, we confirmed the expression of E2Fs in PAAD by IHC.

**Results:**

The transcription levels of E2F1/3/5/8 are obviously up-regulated in PAAD and the high expression of E2F2/3/6/8 was apparently associated with the tumor stage of patients with PAAD. The abnormal expression of E2F1/2/3/4/5/7/8 in PAAD patients is related to the clinical outcome of PAAD patients. We also found that PAAD tissues have higher expression levels of E2F1/3/5/8 compared with adjacent normal tissues. The function of E2Fs and its neighboring genes is mainly related to the transcription initiation of the RNA polymerase II promoter. The functions of E2Fs and its neighboring proteins are mainly related to cell cycle, virus carcinogenesis, FoxO signaling pathway, TGF-*β* signaling pathway, transcriptional disorders in cancer and Wnt signaling pathway. We also found that the expression of E2Fs was significantly correlated with immune infiltrates, including B cells, CD8+ T cells, CD4+T cells, neutrophils, macrophages, and dendritic cells.

**Conclusions:**

Our study may provide new insights into the choice of immunotherapy targets and prognostic biomarkers in PAAD patients.

## Introduction

Pancreatic adenocarcinoma (PAAD) is a malignant tumor of the digestive system that is difficult to diagnose and treat (1). Due to the difficulty of early diagnosis, high surgical mortality, poor treatment outcome, the 5-year survival rate of PAAD patients is only 5%, and it has become one of the tumors with high incidence and malignancy in developed countries (2–4). Although PAAD treatment has been partially improved in recent decades, most patients are diagnosed with cancer only in the middle and late stages of cancer because the early symptoms of PAAD patients are not obvious, and there is a lack of biomarkers for early diagnosis. This situation is not effective for surgical resection and drug treatment and ultimately leads to high mortality in patients with PAAD (5). Therefore, it is urgent to find effective biomarkers for early diagnosis and prognosis of PAAD and develop new strategies for targeted therapies for PAAD.

A total of eight E2Fs have been discovered since the first E2F transcription factor (E2F1) was discovered in mammalian cells (6–13). E2Fs are a group of genes encoding a family of transcription factors in higher eukaryotes that are involved in a variety of cellular functions and are expressed in many tissues and organs (14). According to the different functions of E2Fs, they can be divided into two types: transcription activators (E2F1–E2F3) and transcription repressors (E2F4–E2F8). Some studies have found that E2Fs often play a role downstream of mammalian cell cycle signal transduction and affect and control cell proliferation, differentiation, apoptosis, and aging by regulating many target genes (14–17). E2Fs are abnormally expressed in a variety of human malignancies, such as breast cancer (18), ovarian cancer (19), high-grade glioma (20), liver cancer (21), and pancreatic adenocarcinoma (22). Sun F et al. (22) found that overexpression of E2F3 promoted the proliferation of pancreatic tumor cells and the development of pancreatic adenocarcinoma. Lin C et al. (23) suggest that pancreatic tumor growth could be inhibited by inhibiting the expression of E2F5 by miR-1179 overexpression. However, the expression level, genetic variation, molecular mechanism and biological function of different E2Fs in PAAD and its relationship with the prognosis and immune infiltration in patients with PAAD have not been fully elucidated.

Microarray and RNA sequencing technologies have brought huge changes to gene research, and have gradually become a significant part of biological and biomedical research (24, 25). In this study, we used various large-scale bioinformatics databases to conduct deeply into and comprehensive bioinformatics exploration of the expression of E2Fs in PAAD. The expression and mutation of different E2Fs in PAAD were analyzed to determine the expression pattern, biological function, prognostic value, and relationship with immune infiltration of E2Fs in PAAD.

## Materials and Methods

### Ethics Statement

The study proposal has been approved by the Ethics Committee of Taihe Hospital Affiliated of Hubei University of Medicine (Shiyan, China) (document NO.2020KS042) and conducted in accordance with the principles of the Helsinki Declaration.

### Oncomine Analysis

Oncomine gene expression array dataset (www.oncomine.org) is a publicly accessible online cancer microarray database for analyzing the E2Fs transcription levels in different cancers (26, 27). Student’s t test was used to compare the transcription levels of E2Fs in clinical cancer specimens with that in normal controls. The cut-off of *P*-value and fold change were defined as 0.05 and 1.5, respectively.

### GEPIA Dataset Analysis

Gene Expression Profiling Interactive Analysis (GEPIA, www.gepia.cancer-pku.cn) is a newly developed interactive web server that analyzes RNA sequencing expression data from 9,736 tumors and 8,587 normal samples from the Cancer Genome Atlas (TCGA) and Genotypic Tissue Expression (GTEx) projects using standard processing pipelines (www.gepia.cancer-pku.cn/). GEPIA can provide customizable functions, including differential expression analysis, patient survival analysis, cancer type staging, cancer pathological staging, correlation analysis, similar gene detection and dimensionality reduction analysis (28).

### The Kaplan–Meier Plotter Analysis

The Kaplan–Meier plotter is an online database (www.kmplot.com) that contains gene expression data and survival information for clinical cancer patients (29, 30). We used this online tool to assess the prognostic value of E2Fs mRNA expression in PAAD patients and analyzed the overall survival (OS) and recurrence-free survival (RFS) of patients with PAAD. The database divides patient samples into high expression groups and low expression groups according to the median values of E2Fs mRNA expression and validates them by Kaplan–Meier survival curve. Information on number of patients, median values of mRNA expression, 95% confidence interval (CI), hazard ratio (HR), and *P*-value can be found on the Kaplan–Meier plotter web page. *P*-value <0.05 was considered as statistically significant.

### TCGA Data and cBioPortal

The TCGA database contains sequencing and pathological data for more than 30 different cancers (31). We selected a Pancreatic Adenocarcinoma dataset (TCGA, PanCancer Atlas) that included 184 pathological reports and further analyzed the expression of E2Fs using cBioPortal (www.cbioportal.org) (32, 33). The genomic map includes data for putative copy-number alterations (CNA) from GISTIC, mRNA expression z-scores and mutations. Follow the online instructions of cBioPortal for co-expression and network analysis.

### GeneMANIA Analysis

GeneMANIA (www.genemania.org) is an online analysis tool for deriving hypotheses based on gene functions. GeneMANIA can query and generate a list of genes with similar functions to the target gene and illustrate the relationship between the target gene and the data set by constructing an interactive network (34). In this study, we used GeneMANIA to construct a gene–gene interaction network for E2Fs.

### STRINGS Analysis

STRINGS (www.string-db.org) is an online analysis tool that collects, scores and integrates all publicly available sources of protein–protein interaction (PPI) data and supplements it with calculations and predictions (35). In this study, we performed a PPI network analysis on differentially expressed E2Fs to explore their interactions.

### Metascape Analysis

Metascape (www.genemania.org) is a free and well-maintained online bioinformatics database for Gene Ontology (GO) and Kyoto Encyclopedia of Genes and Genomes (KEGG) enrichment analysis (36). In this study, we used Metascape for functional annotation and pathway enrichment analysis of E2Fs and neighboring genes significantly associated with E2Fs. Only terms with *P*-value <0.01, minimum count >3, and enrichment factor >1.5 were considered as significant.

### TIMER Analysis

TIMER (www.cistrome.shinyapps.io) is a convenient and accurate online analysis tool that can infer the abundance of tumor-infiltrating immune cells from gene expression profiles and evaluate their clinical impact (37). In this study, we used TIMER to assess the correlation between E2Fs expression levels and immune cell infiltration and to assess the correlation between clinical outcomes and immune cell infiltration and E2Fs expression.

### Immunohistochemistry

Clinical samples were obtained from 56 patients with PAAD who were surgically treated at Taihe Hospital Affiliated of Hubei University of Medicine from January 2017 to December 2018. The PAAD tissue and the paired adjacent tissues were prepared into 3 μm paraffin sections and incubated with rabbit polyclonal antibodies of E2F1, E2F2, E2F3, E2F4, E2F5, E2F6, E2F7, and E2F8 (1:150, Abcam, USA) at 4° overnight in a refrigerator. The sections were coupled with secondary antibody labeled with horseradish peroxidase (1:400, Abcam, USA) at room temperature for 1.5 h, then each section was stained with DAB reagent, and finally counterstained with hematoxylin.

## Results

### Transcriptional Levels of E2Fs in PAAD Patients

**Figure 1:**
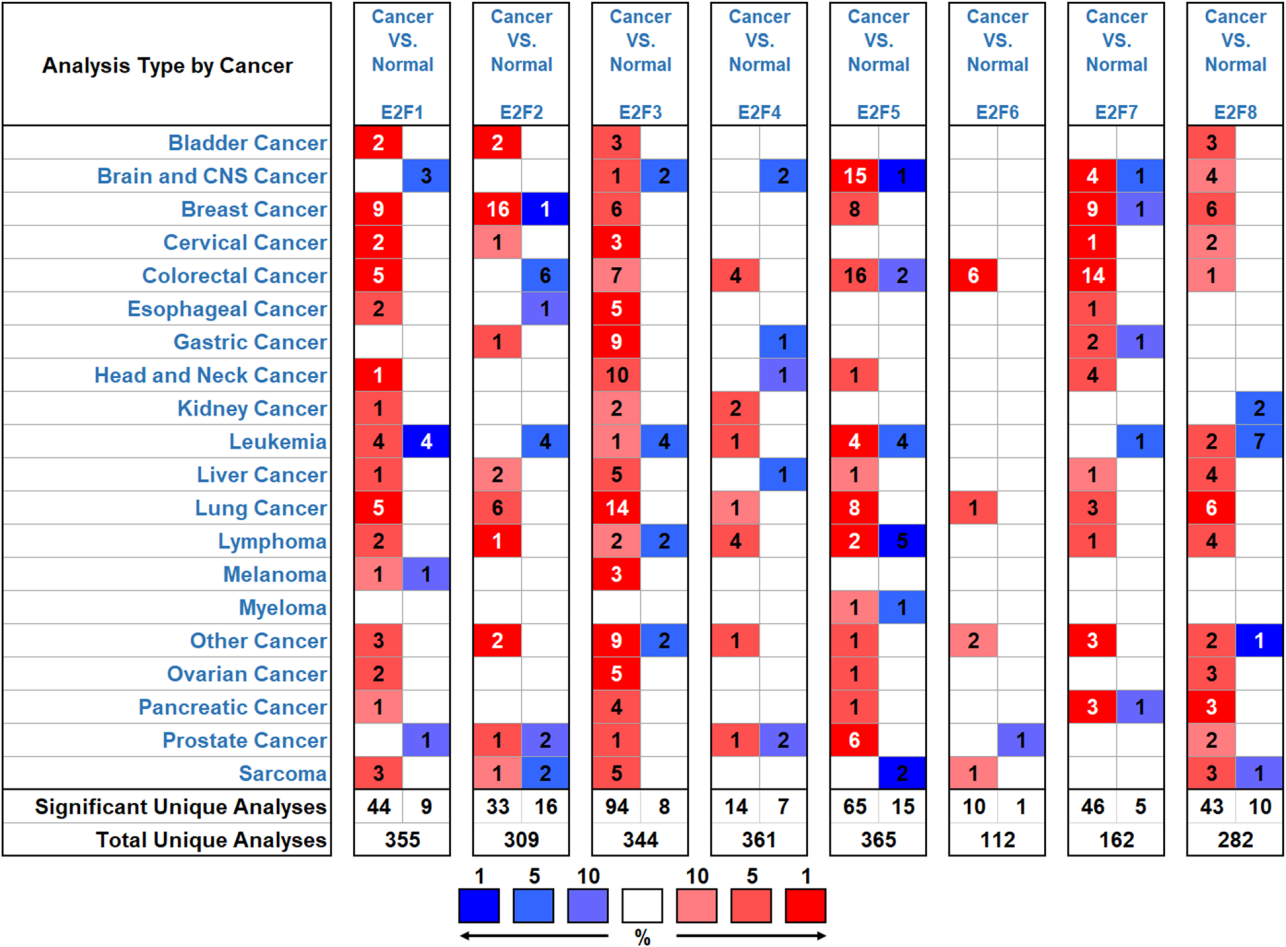
The transcription levels of E2F factors in different types of cancers (Oncomine). The panel shows the numbers of datasets with statistically significant mRNA over-expression (red) or downregulated expression (blue) of E2F factors. The threshold was designed with the following parameters: fold change of 1.5 and *P*-value of 0.05.

Eight E2Fs are widely found in mammalian cells but are expressed abnormally in different tumor tissues. We used the Oncomine database to compare the mRNA expression of E2Fs in different cancer and normal tissue samples (**Figure 1**). ONCOMINE analysis showed that the transcriptional levels of E2F1/3/5/7/8 were up-regulated in PAAD patients. In the Buchholz dataset (38), the transcriptional levels of E2F1 in PAAD were significantly higher than normal tissues, with a fold change of 1.759 (*P* = 0.003). In three datasets, E2F3 transcription levels were significantly up-regulated in PAAD patients. In the Logsdon dataset (39), the expression of E2F3 in the two groups of PAAD was significantly up-regulated compared with normal samples, with fold changes of 1.551 (*P* = 0.001) and 1.963 (*P* = 4.87E-5), respectively (**Table 1**). In the Segara dataset (40), the expression of E2F3 increased in PAAD with a fold change of 1.533 and (*P* = 4.25E-4). In the Badea dataset (41), compared with normal samples, the expression of E2F3 increased in PAAD with a fold change of 2.519 (*P* = 6.33E-9). In the Grutzmann dataset (42), the expression of E2F5/7/8 was significantly increased in PAAD, with fold changes of 1.603 (*P* = 0.009), 4.181 (*P* = 7.70E-4), and 4.334 (*P* = 0.002), respectively. In the Ishikawa (43) and Pei datasets (44), the expression levels of E2F7 and E2F8 in PAAD were significantly higher than normal tissues, with fold changes of E2F7 of 1.766 (*P* = 1.13E-4) and 2.029 (*P* = 2.31E-6), and fold changes of E2F8 of 2.063 (*P* = 0.007) and 3.028 (*P* = 2.67E-8), respectively. In addition, ONCOMINE analysis revealed no significant contrast in the transcriptional levels of E2F2, E2F4, and E2F6 between pancreatic adenocarcinoma and normal samples.

**Table 1:** The Significant Changes of E2F Expression in Transcription Level between Different Types of Pancreatic adenocarcinoma and Normal Pancreatic Tissues (Oncomine).

	Types of PAAD *vs*. normal	Fold change	*t*-test	*P*-value	Ref
E2F1	Pancreatic Ductal Adenocarcinoma	1.759	3.750	0.003	[38]
E2F2	NA	NA	NA	NA	NA
E2F3	Pancreatitis	1.551	4.621	0.001	[39]
	Pancreatic Adenocarcinoma	1.963	5.630	4.87E-5	[39]
	Pancreatic Carcinoma	1.533	4.383	4.25E-4	[40]
	Pancreatic Ductal Adenocarcinoma	2.519	6.677	6.33E-9	[41]
E2F4	NA	NA	NA	NA	NA
E2F5	Pancreatic Ductal Adenocarcinoma Epithelia	1.603	2.572	0.009	[42]
E2F6	NA	NA	NA	NA	NA
E2F7	Pancreatic Ductal Adenocarcinoma	1.766	4.018	1.13E-4	[43]
	Pancreatic Ductal Adenocarcinoma Epithelia	4.181	3.680	7.70E-4	[42]
	Pancreatic Carcinoma	2.029	5.188	2.31E-6	[44]
E2F8	Pancreatic Ductal Adenocarcinoma Epithelia	4.334	3.413	0.002	[42]
	Pancreatic Carcinoma	3.028	6.769	2.67E-8	[44]
	Pancreatic Ductal Adenocarcinoma	2.063	2.543	0.007	[43]

NA, not available.

### Relationship Between the Transcriptional Levels of E2Fs and the Clinicopathological Parameters of PAAD Patients

**Figure 2:**
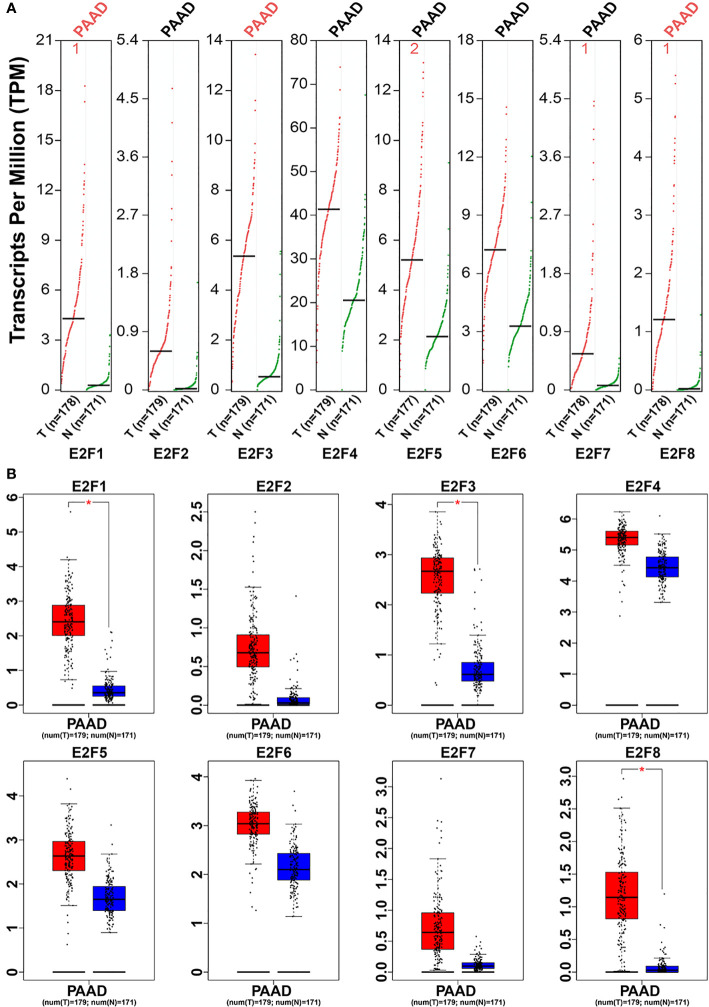
The expression of E2Fs in PAAD (GEPIA).

**Figure 3:**
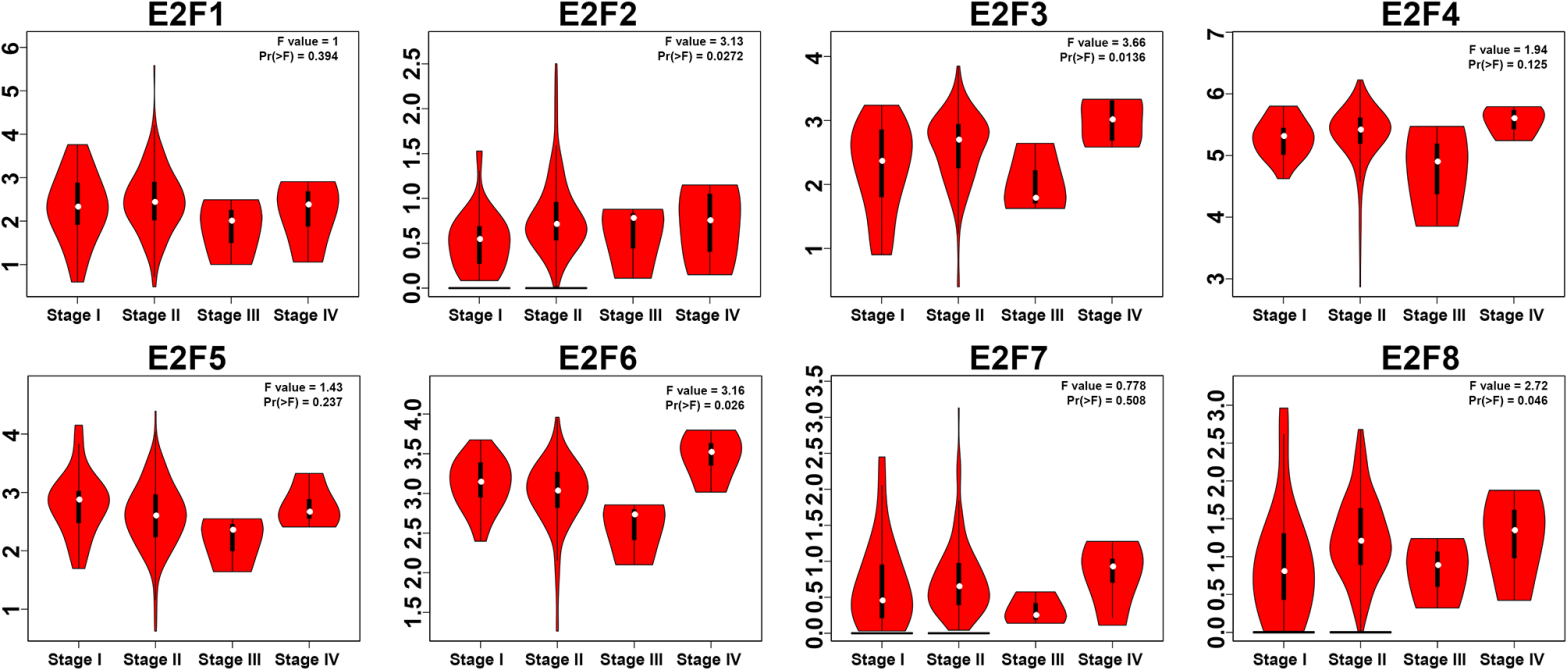
Correlation between E2Fs expression and Tumor Stage in PAAD patients (GEPIA).

We used the GEPIA dataset to compare the transcriptional levels of E2F factors between PAAD and normal simples (**Figure 2**). Studies have shown that the transcriptional levels of E2F1/3/8 in PAAD tissues were significantly higher than normal pancreatic tissues, while the transcriptional levels of E2F2/4/5/6/7 were not significantly contrast between PAAD tissues and normal pancreatic simples. We also studied the relationship between mRNA expression levels of E2Fs and tumor stage in PAAD patients by using the GEPIA dataset. The results showed that the expression levels of E2F2, E2F3, E2F6, and E2F8 are significantly correlated with the tumor stage of PAAD patients, while the expression levels of E2F1, E2F4, E2F5 and E2F7 are not correlated with the tumor stage of PAAD patients. (**Figure 3**).

**Figure 4:**
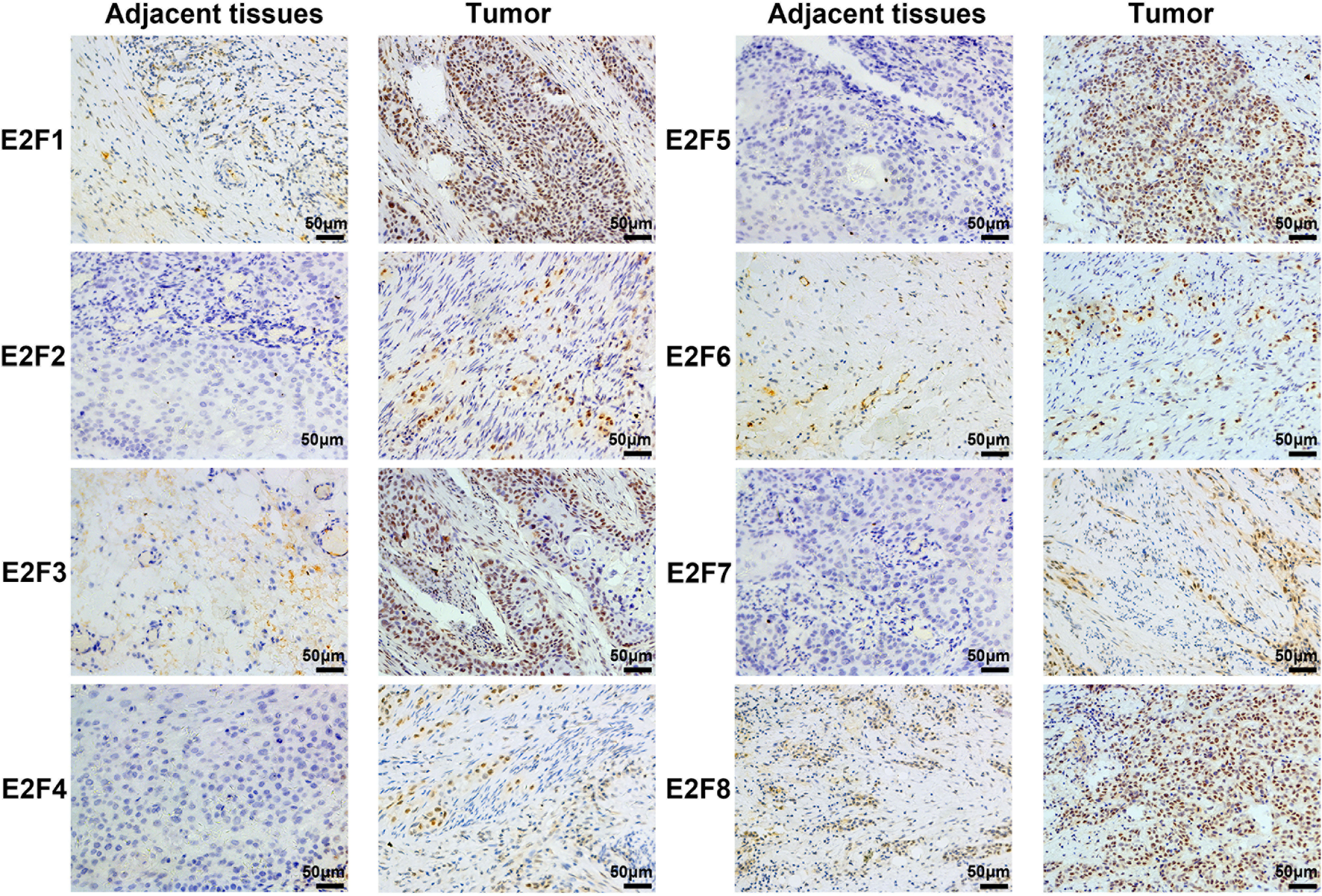
The Expression of E2Fs in PAAD (IHC).

We used immunohistochemistry (IHC) to detect the protein expression of E2Fs in PAAD and its paired adjacent tissues. We found that the protein levels of E2F1, E2F3, E2F5, and E2F8 are higher in PAAD tissues than in the adjacent tissues (**Figure 4**).

### The Prognostic Value of E2Fs in PAAD Patients

**Figure 5:**
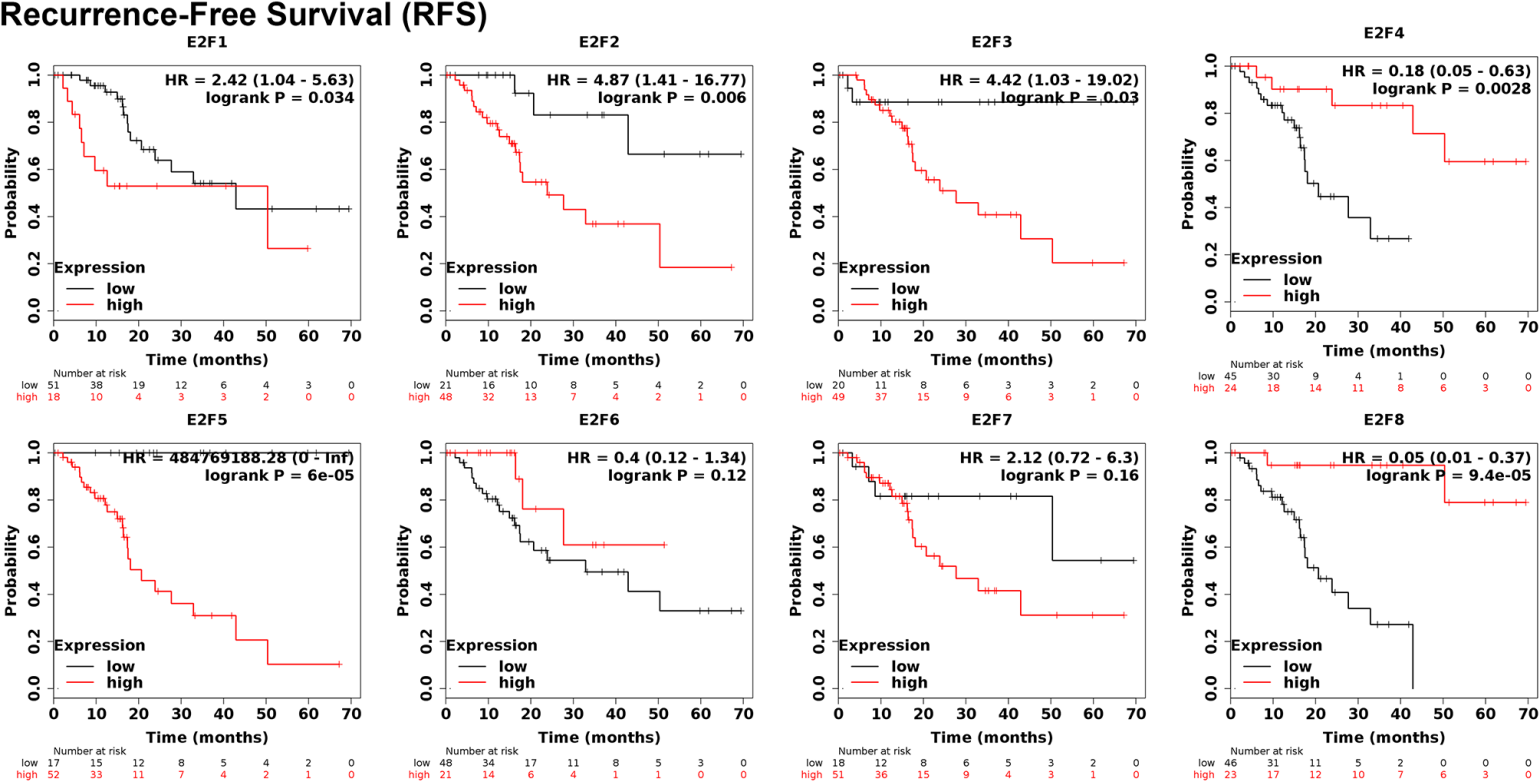
The prognostic value of different expressed E2Fs in PAAD patients in the RFS curve (Kaplan–Meier plotter).

**Figure 6:**
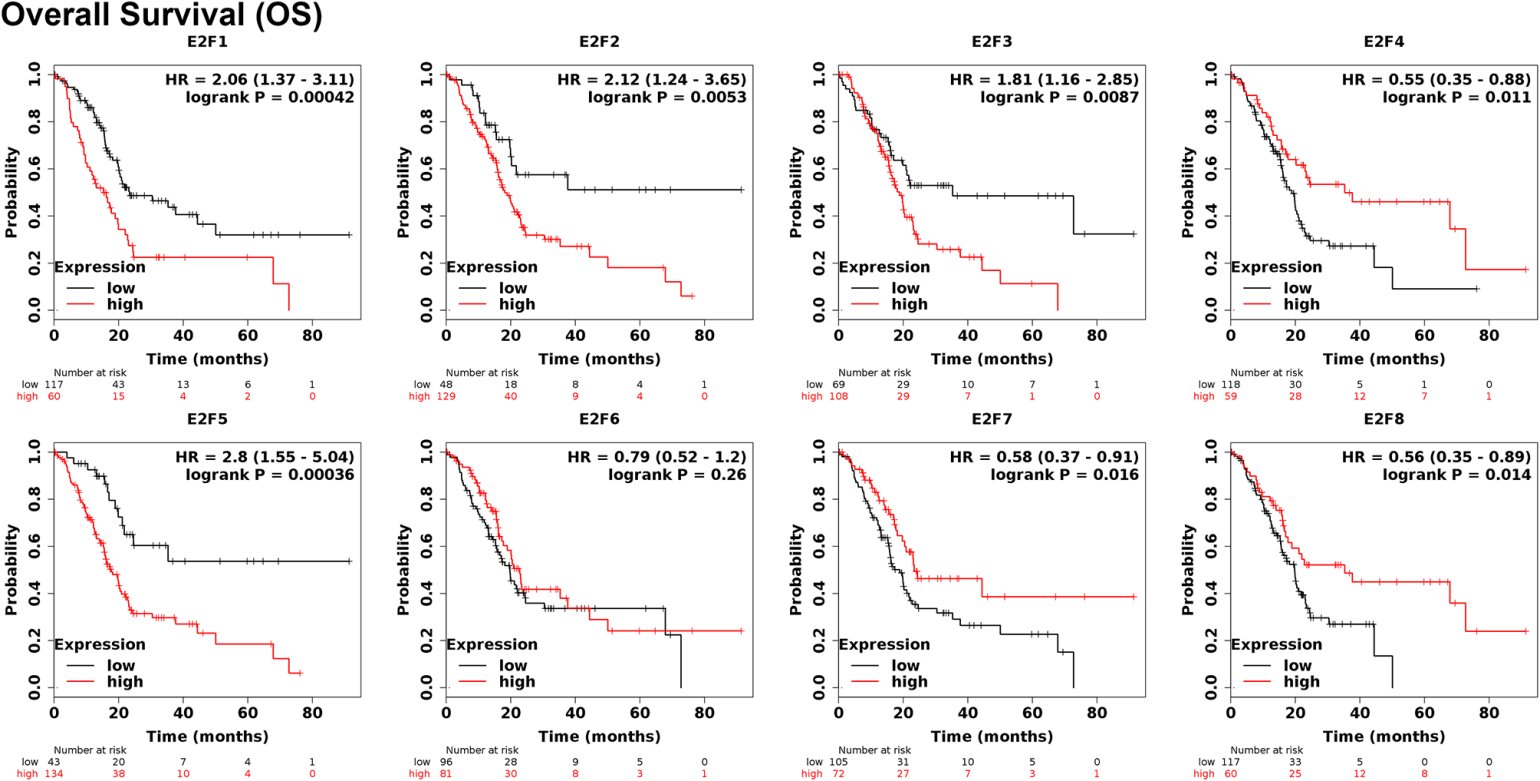
The prognostic value of different expressed E2Fs in PAAD patients in the OS curve (Kaplan-Meier plotter).

To evaluate the value of E2Fs at different transcription levels in the progression of PAAD, we evaluated the correlation between E2Fs at different transcription levels and clinical outcome using Kaplan–Meier plotter analysis. The RFS curve is shown in **Figure 5**. PAAD patients with low transcription levels of E2F1/2/3/5 were significantly associated with longer RFS. In contrast, PAAD patients with high transcription levels of E2F4 and E2F8 were significantly related with longer RFS, while transcription levels of E2F6 and E2F7 were not related to RFS in PAAD patients. The value of E2Fs at different transcription levels in overall survival of PAAD patients was also evaluated. The studies showed that PAAD with low expression of E2F1/2/3/5 was significantly associated with longer OS. In contrast, PAAD patients with high mRNA expression of E2F4/7/8 were significantly related with longer OS, while E2F6 transcription levels were not associated with OS in PAAD patients (**Figure 6**).

### Genetic Alteration, Co-expression, Neighbor Gene Network, and Interaction Analyses of E2Fs in PAAD Patients

**Figure 7:**
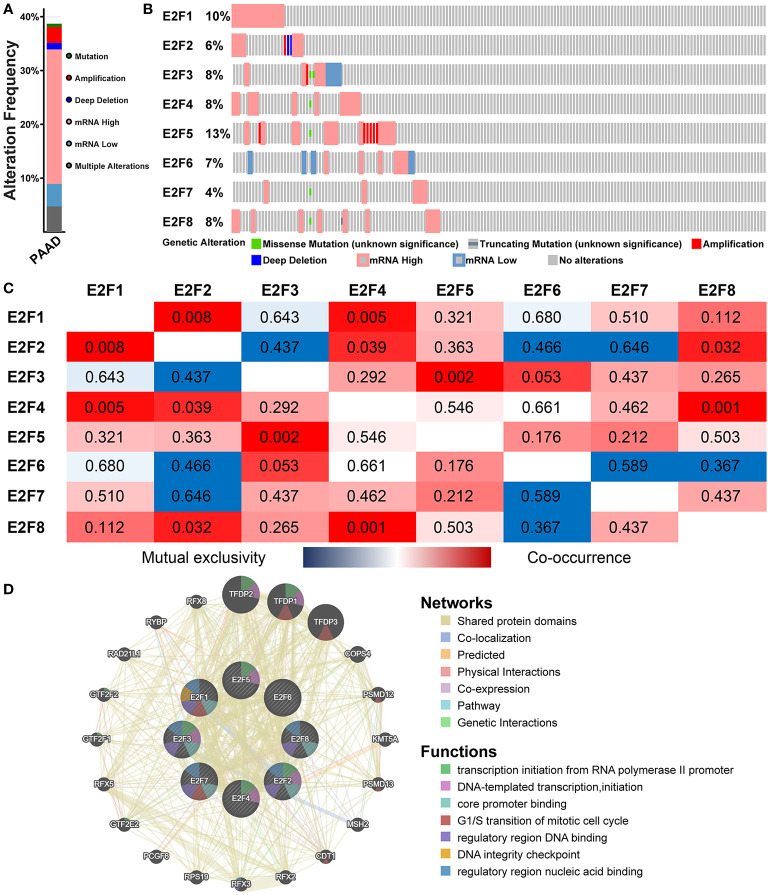
Genetic alteration, co-expression, neighbor gene network, and interaction analyses of E2Fs in patients with PAAD (cBioPortal and GeneMANIA). **(A)** Summary of alterations in different expressed E2Fs in PAAD. **(B)** OncoPrint visual summary of alteration on a query of E2F family members. **(C)** Correlation heat map of different expressed E2Fs in PAAD. Red and blue cells indicate co-occurrence and mutual exclusivity, respectively. The numbers in the color blocks represent the *P*-values. **(D)** Gene–gene interaction network of different expressed E2Fs. Each node represents a gene, and the size of the node represents the strength of the interaction. The color of the connection lines between nodes represents the type of gene–gene interaction. Node colors indicate the possible functions of the respective genes.

We used the cBioPortal online tool to analyze changes and correlations of E2Fs in PAAD. Among 168 PAAD patients, E2Fs were changed in 65 samples (38.69%), and two or more changes were detected in eight samples (4.76%) (**Figure 7A**). According to the TCGA data set, the highest genetic variation rate in E2Fs is E2F5 (10%), the lowest mutation rate is E2F7 (4%), and the others are E2F1 (10%), E2F2 (6%), E2F3 (8%), E2F4 (8%), E2F6 (7%), and E2F8 (8%) (**Figure 7B**). Evaluation of the mutual exclusion between the eight E2Fs genes in the TCGA PAAD cohort showed that there are co-expressed relationships between E2F1 and E2F2, E2F1 and E2F4, E2F2 and E2F4, E2F2 and E2F8, E2F3 and E2F5, E2F4 and E2F8 (*P* < 0.05), E2F2 with E2F3, six, seven and E2F6 with E2F7, eight are highly mutually exclusive but not statistically significant (*P* > 0.05), probably due to the small sample size (**Figure 7C**).

A GGI network of eight E2Fs was constructed, and their functions were analyzed using the GeneMANIA database (**Figure 7D**). The eight central nodes of E2Fs are surrounded by 20 nodes representing genes that are strongly associated with E2Fs in shared protein domains, physical interactions, co-localization, co-expression, prediction, genetic interactions, and pathway. The top five genes most associated with E2Fs are TFDP2 (transcription factor Dp-2), TFDP1 (transcription factor Dp-1), TFDP3 (transcription factor Dp-3), COPS4 (COP9 signalosome subunit 4), and PSMD12 (proteasome 26S subunit, non-ATPase 12). Among them, TFDP2 is related to E2F1,2,3,4,6 in terms of physical interaction and has a pathway relationship with E2F1 and E2F4. TFDP1 is related to E2F1,2,3,4,5,6 in terms of physical interaction, and it is related to genetic interaction in E2F2. However, TFDP2, TFDP1, TFDP3, COPS4, PSMD12, and E2F1/2/3/4/5/6/7/8 all have shared protein domains. Further functional analysis showed that these genes indicated the greatest correlation with transcription initiation from RNA polymerase II promoter (FDR = 7.28E-8). In addition, these genes were correlated with DNA-templated transcription, initiation, core promoter binding, G1/S transition of mitotic cell cycle, regulatory region DNA binding, DNA integrity checkpoint and regulatory region nucleic acid binding activity.

### PPI and Functional Enrichment Analysis of E2Fs in PAAD Patients

**Figure 8:**
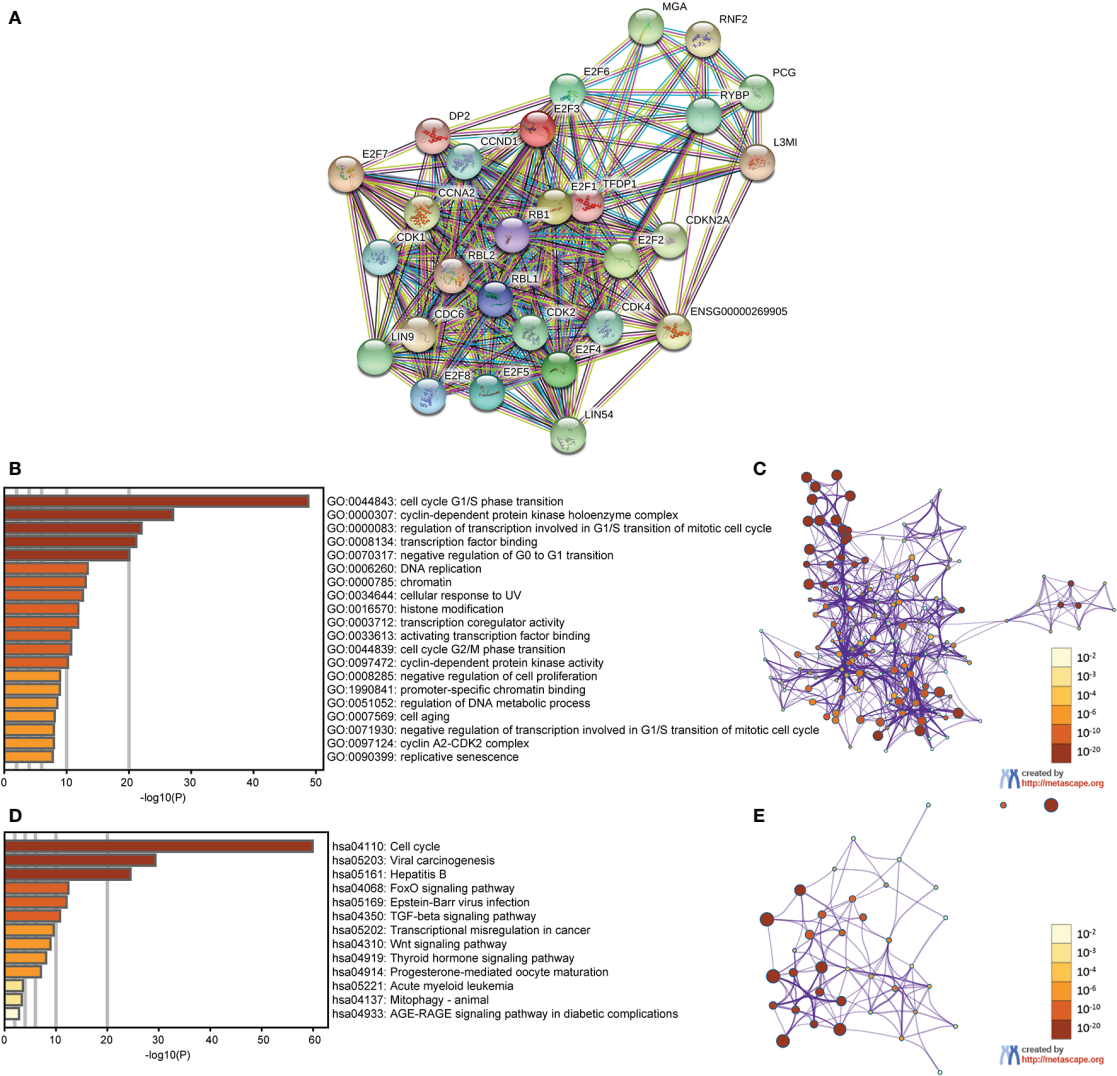
PPI and functional enrichment analysis of E2Fs in patients with PAAD (STRING and Metascape). **(A)** Protein–protein interaction network of different expressed E2Fs. **(B)** E2Fs gene ontology (GO) enriched terms, colored by *P*-values. **(C)** Network of GO enriched terms colored by *P*-value, where terms containing more genes tend to have a more significant *P*-value. **(D)** E2Fs Kyoto Encyclopedia of Genes and Genomes (KEGG) enriched terms, colored by *P*-values. **(E)** Network of KEGG enriched terms colored by *P*-value, where terms containing more genes tend to have a more significant *P*-value.

We performed a PPI network analysis of E2Fs at different transcription levels using STRING to study the potential interactions between them. As shown in **Figure 8A**, the PPI network diagram contains eight E2Fs proteins and 20 proteins that are closely related to E2Fs.

In this study, we used Metascape to perform functional annotation and pathway enrichment analysis of E2Fs and their adjacent genes. The first 20 items of GO enrichment are mainly distributed in the biological process (11 items), the molecular function (six items) and the cell component (three items) (**Figures 8B, C** and **Table 2**). Three of the first five projects are in the biological process, which are cell cycle G1/S phase transition, regulation of transcription involved in G1/S transition of mitotic cell cycle, and negative regulation of G0 to G1 transition, and the other two are cyclin-dependent protein kinase holoenzyme complex and transcription factor binding.

**Table 2:** The GO function enrichment analysis of E2Fs and neighbor genes in PAAD (Metascape).

GO	Category	Description	Count	%	Log10(*P*)	Log10(*q*)
GO:0044843	GO Biological Processes	cell cycle G1/S phase transition	33	60	−48.77	−44.42
GO:0000307	GO Cellular Components	cyclin-dependent protein kinase holoenzyme complex	14	25.45	−27.08	−24.07
GO:0000083	GO Biological Processes	regulation of transcription involved in G1/S transition of mitotic cell cycle	11	20	−22.01	−19.28
GO:0008134	GO Molecular Functions	transcription factor binding	23	41.82	−21.19	−18.5
GO:0070317	GO Biological Processes	negative regulation of G0 to G1 transition	11	20	−20.06	−17.42
GO:0006260	GO Biological Processes	DNA replication	13	23.64	−13.39	−10.89
GO:0000785	GO Cellular Components	chromatin	16	29.09	−13.08	−10.58
GO:0034644	GO Biological Processes	cellular response to UV	9	16.36	−12.62	−10.13
GO:0016570	GO Biological Processes	histone modification	14	25.45	−11.88	−9.42
GO:0003712	GO Molecular Functions	transcription coregulator activity	15	27.27	−11.87	−9.41
GO:0033613	GO Molecular Functions	activating transcription factor binding	8	14.55	−10.75	−8.34
GO:0044839	GO Biological Processes	cell cycle G2/M phase transition	11	20	−10.74	−8.34
GO:0097472	GO Molecular Functions	cyclin-dependent protein kinase activity	6	10.91	−10.22	−7.84
GO:0008285	GO Biological Processes	negative regulation of cell proliferation	14	25.45	−8.95	−6.61
GO:1990841	GO Molecular Functions	promoter-specific chromatin binding	6	10.91	−8.92	−6.59
GO:0051052	GO Biological Processes	regulation of DNA metabolic process	11	20	−8.52	−6.21
GO:0007569	GO Biological Processes	cell aging	7	12.73	−8.08	−5.83
GO:0097124	GO Cellular Components	cyclin A2-CDK2 complex	3	5.45	−7.95	−5.71
GO:0071930	GO Biological Processes	negative regulation of transcription involved in G1/S transition of mitotic cell cycle	3	5.45	−7.95	−5.71
GO:0090399	GO Biological Processes	replicative senescence	4	7.27	−7.77	−5.54

The first 13 KEGG pathways of E2Fs and its adjacent genes are illustrated in **Figures 8D, E** and **Table 3**. Among them, cell cycle, transcriptional misregulation in cancer, FoxO signaling pathway, TGF-beta signaling pathway, viral carcinogenesis, and Wnt signaling pathway are significantly associated with the occurrence and development of various tumors and are also involved in the tumorigenesis of PAAD.

**Table 3:** The KEEG function enrichment analysis of E2Fs and neighbor genes in PAAD (Metascape).

GO	Category	Description	Count	%	Log10(*P*)	Log10(*q*)
hsa04110	KEGG Pathway	Cell cycle	32	58.18	−59.93	−57.24
hsa05203	KEGG Pathway	Viral carcinogenesis	21	38.18	−29.32	−26.93
hsa05161	KEGG Pathway	Hepatitis B	17	30.91	−24.49	−22.4
hsa04068	KEGG Pathway	FoxO signaling pathway	10	18.18	−12.4	−10.96
hsa05169	KEGG Pathway	Epstein-Barr virus infection	11	20	−12.06	−10.67
hsa04350	KEGG Pathway	TGF-beta signaling pathway	8	14.55	−10.79	−9.42
hsa05202	KEGG Pathway	Transcriptional misregulation in cancer	9	16.36	−9.56	−8.23
hsa04310	KEGG Pathway	Wnt signaling pathway	8	14.55	−8.92	−7.61
hsa04919	KEGG Pathway	Thyroid hormone signaling pathway	7	12.73	−8.08	−6.79
hsa04914	KEGG Pathway	Progesterone-mediated oocyte maturation	6	10.91	−7.08	−5.8
hsa05221	KEGG Pathway	Acute myeloid leukemia	3	5.45	−3.57	−2.43
hsa04137	KEGG Pathway	Mitophagy - animal	3	5.45	−3.36	−2.24
hsa04933	KEGG Pathway	AGE-RAGE signaling pathway in diabetic complications	3	5.45	−2.82	−1.74

### The Relationship Between E2Fs Expression Levels and Immune Infiltration Levels in PAAD

**Figure 9:**
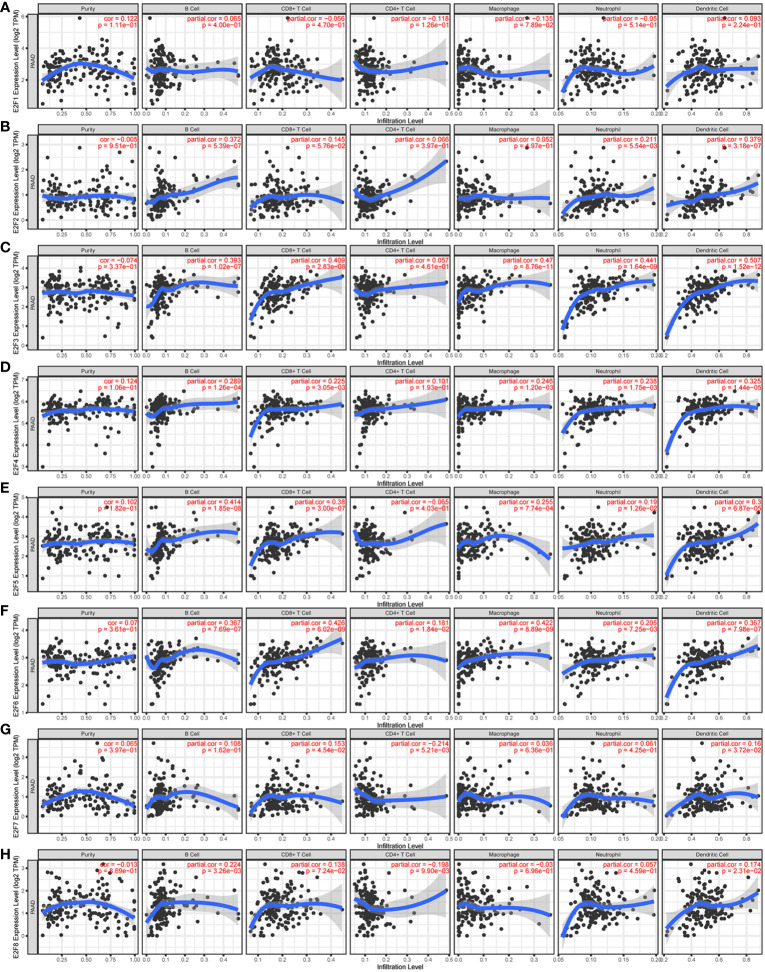
The Relationship between E2Fs Expression Levels and Immune Infiltration Levels in PAAD (TIMER). The correlation between the abundance of immune cell and the expression of **(A)** E2F1, **(B)** E2F2, **(C)** E2F3, **(D)** E2F4, **(E)** E2F5, **(F)** E2F6, **(G)** E2F7, **(H)** E2F8 in PAAD.

TIMER online analysis tool is used to evaluate the relationship between the transcription level of E2Fs and the level of immune infiltration in PAAD. It was found that E2Fs are involved in inflammatory response and immune cell infiltration, which affect the clinical outcome of PAAD patients. The analysis results are shown in **Figure 9**. E2F2 expression was positively correlated with infiltration of B cells, neutrophil, and dendritic cells (**Figure 9B**). E2F3, E2F4, E2F5, and E2F6 expressions were positively correlated with the infiltration of B cells, CD8+ T cells, macrophage, neutrophil, and dendritic cells, and E2F6 expressions were also positively correlated with the infiltration of CD4+ T cells (**Figures 9C–F**). E2F7 expression was positively correlated with infiltration of CD8+ T cells and dendritic cells, while it was negatively correlated with infiltration of CD4+ T cells (**Figure 9G**). E2F8 expression was negatively correlated with infiltration of CD4+ T cells, while it was positively correlated with infiltration of B cells and dendritic cells (**Figure 9H**). These studies indicate that the level of E2Fs expression is associated to the level of immune infiltration in PAAD.

We also use the TIMER online tool to proofread B cells, CD8+ T cells, CD4+ T cells, macrophage, neutrophil, E2F1/2/3/4/5/6/7/8 covariate factors and automatically output Cox regression results. These studies indicate that the infiltration of CD8+ T cells (*P* = 0.030), neutrophil (*P* = 0.026), and E2F1 (*P* = 0.015) was significantly correlated with the clinical outcomes of patients with PAAD (**Table 4**).

**Table 4:** The cox proportional hazard model of CXC chemokines and six tumor-infiltrating immune cells in PAAD (TIMER).

	coef	HR	95%CI_l	95%CI_u	*P*-value	sig
B_cell	3.833	46.203	0.193	1.11E+04	0.170	
CD8_Tcell	7.455	1,728.250	2.090	1.43E+06	0.030	*
CD4_Tcell	−2.800	0.061	0.000	6.44E+01	0.431	
Macrophage	−5.591	0.004	0.000	1.32E+00	0.062	
Neutrophil	15.292	437,7762.585	6.027	3.18E+12	0.026	*
Dendritic	−1.940	0.144	0.005	4.29E+00	0.263	
E2F1	0.576	1.780	1.120	2.83E+00	0.015	*
E2F2	−0.481	0.618	0.301	1.27E+00	0.189	
E2F3	0.188	1.207	0.704	2.07E+00	0.494	
E2F4	−0.578	0.561	0.252	1.25E+00	0.157	
E2F5	−0.126	0.882	0.548	1.42E+00	0.604	
E2F6	−0.695	0.499	0.223	1.12E+00	0.091	
E2F7	0.143	1.154	0.733	1.82E+00	0.535	

*P < 0.05.

## Discussion

Studies have shown that the dysregulation of E2Fs is significantly associated to the occurrence and development of many tumors (18–23). E2Fs not only participate in tumor cell proliferation and differentiation, but also affect cell apoptosis and senescence (14–17). Although the role of E2Fs in the occurrence and survival prognosis of certain cancers has been elucidated, there have been no reports of different E2Fs expression and role in PAAD. This study is the first study to explore the transcription level, genetic variation, molecular mechanism, and biological function of different E2Fs in PAAD and its relationship with the prognosis and immune infiltration in patients with PAAD through bioinformatics analysis.

Among all members of E2Fs, E2F1 is the earliest found and most researched member. Studies have shown that E2F1 can play different roles in tumor suppressor genes or oncogenes in different cancers (45, 46). However, Ma L et al. (47) found that E2F1 is overexpressed in pancreatic adenocarcinoma tissues, and the up-regulation of E2F1 promotes the proliferation of cancer cells and exerts carcinogenesis. In this study, database analysis found that the transcription levels of E2F1 in human PAAD were higher than in normal tissues, and immunohistochemical staining also verified this result. But the expression of E2F1 in PAAD patients has nothing to do with the tumor stage. Kaplan–Meier plotter analysis revealed that high E2F1 transcription levels were associated with poor OS and RFS in PAAD patients.

E2F2 plays a significant role in the occurrence and development of PAAD. E2F2, like E2F1, can play a dual role in suppressing and causing cancer (48, 49). Yao Z et al. (50) found that miR-214-5p can regulate the expression of E2F2 in PAAD cells to achieve the role of oncogenes. However, in this study, database analysis found no difference in E2F2 expression in PAAD compared to normal tissues. However, the expression of E2F2 in patients with PAAD is related to the stage of the tumor. It was also found that the high transcription levels of E2F2 was related with poor OS and RFS in PAAD patients.

E2F3 is crucial for controlling tumor cell proliferation rate and regulating cell cycle (8). Sun FB et al. (22) found that up-regulating E2F3 expression by knocking down MiR-210 promotes tumor cell proliferation and thus the development of pancreatic adenocarcinoma. The analysis of the data and IHC in this study found that the expression levels of E2F3 in human PAAD were higher than in normal tissues, and it was related to tumor stage in PAAD patients. The results of survival analysis indicated that high transcription levels of E2F3 result in worse OS and RFS in patients with PAAD.

E2F4 is an important regulator of cell transformation and proliferation. Studies have shown that over-expressed E2F4 is related to colon cancer, kidney cancer, and lung cancer. However, the analysis of the data in this study found that the transcription levels of E2F4 in PAAD were not different from that in normal tissues and had nothing to do with the tumor stage of PAAD patients. The results of survival analysis indicated that high expression of E2F4 was associated with better OS and RFS in PAAD patients. This result may herald the role of E2F4 as a tumor suppressor gene in PAAD.

E2F5 is an important member of E2Fs. E2Fs have been studied more in other tumors but less in PAAD. In this study, it was found that the expression level of E2F5 in human PAAD is significantly different from that of normal tissues, but the expression level has nothing to do with the tumor stage of PAAD patients. However, the results of survival analysis indicated that high transcription levels of E2F5 lead to worse OS and RFS in PAAD patients.

No article on the role of E2F6 in PAAD has been published. The results from our analysis found that although E2F6 is related to tumor stage in PAAD patients, the expression of E2F6 is not different between PAAD and normal tissues and has no effect on the survival prognosis of PAAD.

Some studies indicate that E2F7 is up-regulated in many tumors, but there is no relevant report on the role of E2F7 in PAAD. The analysis of this study found that the transcription levels of E2F7 were not different between PAAD and normal tissues, and had no effect on tumor stage. However, survival analysis showed that high expression of E2F7 leads to better OS in patients with PAAD.

As for E2F8, although its role in PAAD has not been reported, our study found that the transcription level of E2F8 in PAAD is significantly higher than in normal tissues, and it is closely related to the tumor stage of PAAD patients. However, survival analysis showed that high transcription level of E2F8 leads to worse OS and RFS in PAAD patients.

Furthermore, a high mutation rate (38.69%) of E2Fs was noticed in PAAD patients, and a mutually exclusive or co-occurring connection was found between differentially expressed E2Fs, indicating that these cytokines play an antagonistic or synergistic role in the occurrence and development of PAAD. We also constructed a GGI network of E2Fs and its neighboring genes, and found that these genes showed the greatest correlation with the transcription initiation of the RNA polymerase II promoter. Then we analyzed the function of E2Fs and its neighboring proteins by GO enrichment analysis and KEGG pathway enrichment. Studies have found that the functions of these proteins are mainly related to the cell cycle, viral carcinogenesis, FoxO signaling pathway, TGF-beta signaling pathway, transcriptional misregulation in cancer and Wnt signaling pathway, and these pathways are significantly associated with the occurrence and development of PAAD. These data indicate that differentially expressed E2Fs in PAAD are potential targets for drug therapy.

Another important result of this study is that the transcription levels of E2Fs is closely correlated with various levels of immune infiltration in PAAD. The transcription level of E2F2 was positively correlated with infiltration of B cells, neutrophil, and dendritic cells. E2F3, E2F4, E2F5, and E2F6 expressions were positively correlated with the infiltration of B cells, CD8+ T cells, macrophage, neutrophil, and dendritic cells, and E2F6 expressions were also positively correlated with the infiltration of CD4+ T cells. The expression level of E2F7 was positively correlated with infiltration of CD8+ T cells and dendritic cells, while it was negatively correlated with infiltration of CD4+ T cells. The expression level of E2F8 was negatively correlated with infiltration of CD4+ T cells, while it was positively correlated with infiltration of B cells and dendritic cells. The correlation between the expression of E2Fs and the marker genes of PAAD immune cells indicates that E2Fs may be involved in the regulation of PAAD tumor immunity.

In conclusion, our research indicates that the transcription levels of E2F1/3/5/8 are obviously up-regulated in PAAD and may play a significant role in the occurrence and development of PAAD. The high expression of E2F2/3/6/8 can also be used as molecular markers to classify tumor stages in PAAD patients. In addition, abnormal expressions of E2F1/2/3/4/5/7/8 were found to be related with clinical outcomes in patients with PAAD. The study found that E2F1/2/3/5 can be used as potential survival prognostic biomarkers and targets for PAAD. However, the significant correlation between the expression of E2Fs and the infiltration of the six immune cell types in PAAD: B cells, CD8+ T cells, CD4+ T cells, macrophages, neutrophils, and dendritic cells, suggests that E2Fs may be involved in the regulation of PAAD tumor immunity. It shows that E2Fs not only can be used as a prognostic indicator of patients with PAAD, but also reflect their immune status. These findings may help better study the molecular basis of PAAD, and may help create more appropriate prognostic tools for PAAD and facilitate the development of new immunotherapeutic. However, more experimental studies are needed to confirm our results, thereby promoting the clinical application of E2Fs as a prognostic indicator or immunotherapy target in PAAD.

## Data Availability Statement

The datasets presented in this study can be found in online repositories. The names of the repository/repositories and accession number(s) can be found in the article/supplementary material.

## Ethics Statement

The studies involving human participants were reviewed and approved by Medical Ethics Committee of the Hubei University of Medicine-affiliated Taihe Hospital. Written informed consent for participation was not required for this study in accordance with the national legislation and the institutional requirements.

## Author Contributions

X-SL, YG, and CL conceived the project and wrote the manuscript. X-SL, X-QC, L-MZ, and J-WY participated in data analysis. CL, YG, and X-YK participated in discussion and language editing. Z-JP reviewed the manuscript. All authors contributed to the article and approved the submitted version.

## Funding

This work was supported by the Hubei province’s Outstanding Medical Academic Leader program, the Foundation for Innovative Research Team of Hubei Provincial Department of Education T2020025, the Hubei Provincial Department of Science and Technology Innovation Group Program (grant no. 2019CFA034), Free-exploring Foundation of Hubei University of Medicine (grant no. FDFR201903), Open Project of Hubei Key Laboratory of Embryonic Stem Cell Research (grant no.2020ESOF009) and the Key Discipline Project of Hubei University of Medicine.

## Conflict of Interest

The authors declare that the research was conducted in the absence of any commercial or financial relationships that could be construed as a potential conflict of interest.
